# Commentary: On the need for metaphysics in psychedelic therapy and research

**DOI:** 10.3389/fpsyg.2023.1341566

**Published:** 2024-01-05

**Authors:** Katherine Cheung, David B. Yaden

**Affiliations:** ^1^Department of Bioethics, New York University, New York, NY, United States; ^2^Department of Psychiatry and Behavioral Sciences, Johns Hopkins University School of Medicine, Baltimore, MD, United States

**Keywords:** metaphysics, psychedelics, bioethics, psychedelic-assisted therapy, philosophy

## Introduction

Psychedelic-assisted therapy (PAT) is often accompanied by belief changes—notably, changes in beliefs about the nature of reality may occur. As one such example, individuals may initially believe in naturalism but come to endorse non-naturalistic beliefs such as dualism or theism after PAT. Addressing this possibility, Sjöstedt-Hughes (2023) proposes the integration of metaphysics—the branch of philosophy concerned with the fundamental nature of reality-into PAT as an optional addition. Similarly, Gładziejewski (2023) has argued that metaphysics is inescapable within the context of PAT. How might we best address these metaphysical belief changes in the clinical setting? In this commentary, we respond to Sjöstedt-Hughes' arguments for the optional inclusion of metaphysics in PAT, and advocate for a pluralistic sense of secularism in the medical domain, which may entail not providing such a menu of metaphysical options.

## Metaphysics and evidence of belief change

Akin to Sjöstedt-Hughes, we acknowledge that the issue of metaphysical belief changes in clinical settings is one in need of addressing, and that such belief changes do arise. A number of studies have found evidence of these changes: Timmermann et al. (2021) found that psychedelics can alter metaphysical beliefs, with their results revealing belief shifts away from physicalist views and toward panpsychism and fatalism post-use. Similarly, a study from Nayak et al. found that a single psychedelic experience increased a range of non-physicalist beliefs (Nayak et al., 2023b). However, a recent longitudinal study from Nayak et al. offers evidence that the issue of metaphysical belief change may not be as pressing as originally conceived, with their study finding little to no change in metaphysical beliefs, contrasting the results of previous cross-sectional studies (Nayak et al., 2023a). Nonetheless, the question of how metaphysical belief change should be addressed in PAT is still one well worth exploring, as such belief change can and does occur.

## Discussion

To briefly summarize, Sjöstedt-Hughes proposes that there is a “potential extra benefit to patients in PAT if they are provided with an optional, additional, and intelligible schema and discussion of metaphysical options” during integration (Sjöstedt-Hughes, 2023). He argues that these benefits may came about as patients will have less reason to dismiss their experience as delusional once they realize that each metaphysical position has an established history, and that an optional metaphysics integration would amplify the significance of the therapeutic experience (given that the psychedelic experiences which appear to have the most therapeutic efficacy are metaphysical experiences) (Sjöstedt-Hughes, 2023).

In addressing these metaphysical belief changes, it is important to note that physicians should not push a metaphysical (or religious) belief system onto their patients, nor should they attempt to influence a patient's metaphysical beliefs in any medical setting. This is clearly articulated in the World Psychiatric Association (WPA) Position Statement on Religion and Spirituality, which states that “psychiatrists should not use their professional position for proselytizing for spiritual or secular worldviews” (Moreira-Almeida et al., 2016). However, patients may initiate discussions of metaphysical belief change, and seek guidance on this topic during PAT — how should this be addressed?

As one prong of his argument, Sjöstedt-Hughes conjectures an optional metaphysics integration would amplify the significance of the therapeutic experience (Sjöstedt-Hughes, 2023). However, one should also take into consideration that this has been argued against by Letheby, who presents a compelling counterargument against the metaphysical belief theory of psychedelic efficacy (see Chapter 7, Letheby, 2021). The metaphysical belief theory suggests that the primary therapeutic mechanism of psychedelics involves belief changes toward non-naturalism. Although we are unable to describe his arguments in depth here, Letheby refutes this view and proposes that metaphysical belief change can be viewed as a side-effect of PAT, but not necessary for it. He additionally suggests that through a lens of alternations of the self, physicians can discuss these belief changes without placing them within a metaphysical framework (Letheby, 2021). Regarding the theory, it is additionally uncertain as to whether the psychedelic experience can provide evidence for metaphysical positions: in his seminal book “The varieties of religious experience,” James argues that mystical-type experiences cannot be said to provide evidence for non-naturalistic beliefs, and that such mystical experiences can be considered without considering their source, e.g., non-naturalistic or from one's brain (James, 1902; Yaden and Newberg, 2022).

Sjöstedt-Hughes (2023) suggests that another reason to include metaphysics is that there may be less reason for the individual to reject the significance of their psychedelic experiences as delusional if they realize that each metaphysical position has an established history. Although recognizing the history of each metaphysical framework may be helpful, it is also worth considering that many of the metaphysical positions may be highly technical and alien to most individuals, possibly having the opposite effect than Sjöstedt-Hughes proposes. Moreover, although belief changes do happen, they are often of a relatively small magnitude: emphasizing them through a menu of metaphysical options, even if optional, may amplify these changes more than what would have happened otherwise. Another thread to explore may be whether individuals want to place their experience within an established metaphysical position—some studies of contemporary metaphysical beliefs suggest that individuals often do not want to belong to one group, instead borrowing from different practices and combining them (MacGregor, 2022).

Finally, as Sjöstedt-Hughes (2023) notes, it should be acknowledged that the psychologists, psychiatrists and other counselors involved in PAT have not often been trained in metaphysics. Following the work of Palitsky et al. (2023) on the incorporation of spiritual, existential, religious, and theological (SERT) components in PAT, we advocate for belief changes of this nature to be addressed in a “in a systematized, non-sectarian (ie, pluralistic), and scalable way” from individuals who have been trained in spiritual and religious competencies (Vieten and Lukoff, 2022), although we agree with Sjöstedt-Hughes (2023) that philosophers have an important role in such discussions about clinical competencies related to metaphysical belief changes as well. Such a pluralistic and systematized manner may involve responding to metaphysical belief changes when they emerge and are brought up by the patients, rather than providing a list of metaphysical beliefs. The presentation of a menu of metaphysical options for a patient to endorse may run the risk of imposing certain worldviews, or may inadvertently pressure them to place their experience within a metaphysical framework. To address these belief changes, clinicians may instead focus on supporting their patient in a more general way to emphasize the relevance of the experience to their own life and sense of self, and allow the patient to process their experience and contemplate their own conclusions regarding their belief changes, rather than emphasizing the metaphysical beliefs.

## Author contributions

KC: Conceptualization, Writing – original draft, Writing – review & editing. DY: Conceptualization, Writing – original draft, Writing – review & editing.
